# Doctor—your septic patients have scurvy!

**DOI:** 10.1186/s13054-018-1950-z

**Published:** 2018-01-29

**Authors:** Paul E. Marik, Michael H. Hooper

**Affiliations:** Division of Pulmonary and Critical Care Medicine, Eastern Virginia Medical School, 825 Fairfax Av, Suite 410, Norfolk, VA 23507 USA

Scurvy is a disease of antiquity described in Egyptian Hieroglyphics and responsible for the deaths of thousands of sailors during the Renaissance. Today, clinicians consider scurvy a very rare disease seen only in patients with extreme dietary deficiencies. They would undoubtedly be shocked to learn that about 40% of the patients in their ICU with septic shock have serum levels of vitamin C supporting a diagnosis of scurvy (<11.3 u/mol/l). The remainder of their patients with sepsis are likely to have hypovitaminosis C (serum level < 23 u/mol/l). Half of their nonseptic ICU patients also have hypovitaminosis C. These are the findings recently reported by Carr et al. [1]. Surprisingly, these astonishing observations are not new. It has been known for over two decades that acute illness results in an acute deficiency of vitamin C with low serum and intracellular levels [2–4]. Low plasma concentrations of vitamin C are associated with more severe organ failure and increased risk of mortality [5]. The most likely explanation for the acute vitamin C deficiency (acute scurvy) in patients with sepsis (and other critical illnesses) is a consequence of metabolic consumption [1]. The fall in serum and cellular levels occurs too rapidly to be explained by decreased gastrointestinal absorption or increased urinary losses. Indeed, in a guinea pig model, myocardial ascorbate was depleted within hours of endotoxin administration [6].

Most clinicians are likewise unaware that primates and guinea pigs are the only mammals that are unable to synthesize vitamin C (in their livers) and that all other mammals increase the synthesis of vitamin C during stress (vitamin C is a true stress hormone). Anthropoid primates and guinea pigs have lost the ability to synthesize vitamin C due to mutations in the l-gulono-γ-lactone oxidase (GULO) gene which codes for the enzyme responsible for catalyzing the last step of vitamin C biosynthesis [7]. The inability to synthesize vitamin C may partly explain why humans and guinea pigs have an increased vulnerability to sepsis and to dying from sepsis [8].

The inability to generate vitamin C makes humans very susceptible to dysfunction in a variety of biochemical pathways that are vital for surviving a critical illness such as sepsis. In experimental models of sepsis, treatment with vitamin C limited the deleterious consequences of sepsis by multiple mechanisms, including attenuation of the proinflammatory response, enhancement of the endothelial and epithelial barrier function, and prevention of sepsis-associated coagulation abnormalities [9, 10]. Sepsis is characterized by the excessive production of reactive oxygen species (ROS) by the induction of enzymes such as nicotinamide adenine dinucleotide phosphate-oxidase (NOX) and the uncoupling of mitochondrial oxidative phosphorylation and inducible nitric oxide synthetase [11]. Vitamin C is a key cellular antioxidant which counteracts these ROS. In addition, vitamin C recycles other antioxidants including α-tocopherol (vitamin E) and tetrahydrobiopterin (BH_4_). BH_4_ plays a critical role in the function of endothelial nitric oxide synthase (eNOS). Vitamin C deficiency results in the incomplete regeneration of BH_4_ resulting in the uncoupling of eNOS and the generation of superoxide and peroxynitrite [11]. Vitamin C inhibits activation of nuclear factor kappa-B (NF-κB), a major nuclear transcription factor involved in release of numerous proinflammatory mediators [12]. Vitamin C is an essential cofactor for the activity of monooxygenase and dioxygenase enzymes, including those enzymes required for the synthesis of catecholamines and vasopressin [13]. In addition, vitamin C binds adrenergic receptors increasing catecholamine sensitivity (acts like a vasopressor agent).

These facts provide the scientific underpinning for treating septic patients with intravenous vitamin C. Due to severe total body depletion of vitamin C, the need for rapid correction, and limited gastrointestinal absorption (due to the saturable vitamin C transporter), vitamin C must be given intravenously in an adequate dose [14]. Based on our experience in treating over 300 patients with severe sepsis and septic shock, we believe that a dose of 1.5 g every 6 hours is adequate. In our experience, this dose is remarkably safe without any discernible side effects [1]. We routinely monitor serum oxalate in high-risk patients (kidney transplant, patients with kidney stones, etc); two patients were noted to have increased baseline oxalate levels, both of which fell during treatment with intravenous vitamin C. We ascribe this finding to improved renal function as well as the effect of thiamine on oxalate metabolism. We believe the clinical benefit of vitamin C in patients with sepsis is synergistically enhanced with the addition of low-dose corticosteroids and thiamine [15, 16]. This novel therapeutic intervention is being tested prospectively in a number of ongoing randomized controlled trials (ClinicalTrials.gov NCT03333278, NCT03335124, NCT03258684, NCT 03380507). In addition, VICTAS (Vitamin C, Thiamine and Steroids in Sepsis: A Randomized, Double-Blind, Parallel Group Study in Critically Ill Patients with Sepsis) is a large multicenter study being conducted in the USA that should elucidate the potential benefit of this treatment strategy.
